# Solving the task variant allocation problem in distributed robotics

**DOI:** 10.1007/s10514-018-9742-5

**Published:** 2018-04-25

**Authors:** José Cano, David R. White, Alejandro Bordallo, Ciaran McCreesh, Anna Lito Michala, Jeremy Singer, Vijay Nagarajan

**Affiliations:** 1 School of Informatics, University of Edinburgh, Edinburgh, EH8 9AB UK; 2 School of Computing Science, University of Glasgow, Glasgow, G12 8RZ UK; 3 Department of Computer Science, University College London, London, WC1E 6BT UK

**Keywords:** Task allocation, Distributed robotics, Multi-robot systems, Multi-objective optimisation

## Abstract

We consider the problem of assigning software processes (or tasks) to hardware processors in distributed robotics environments. We introduce the notion of a *task variant*, which supports the adaptation of software to specific hardware configurations. Task variants facilitate the trade-off of functional quality versus the requisite capacity and type of target execution processors. We formalise the problem of assigning task variants to processors as a mathematical model that incorporates typical constraints found in robotics applications; the model is a constrained form of a multi-objective, multi-dimensional, multiple-choice knapsack problem. We propose and evaluate three different solution methods to the problem: constraint programming, a constructive greedy heuristic and a local search metaheuristic. Furthermore, we demonstrate the use of task variants in a real instance of a distributed interactive multi-agent navigation system, showing that our best solution method (constraint programming) improves the system’s quality of service, as compared to the local search metaheuristic, the greedy heuristic and a randomised solution, by an average of 16, 31 and 56% respectively.

## Introduction

Modern robotics systems are increasingly distributed, heterogeneous and collaborative, incorporating multiple independent agents that communicate via message passing and distributed protocols. A distributed approach can offer desirable qualities such as improved performance. Heterogeneity refers to the type and amount of hardware resources (e.g. sensors, CPU capacity) available on each agent in the system. In such systems, the efficient allocation of software processes (referred to as *tasks*) to hardware processors is of paramount importance in ensuring optimality. Previous works (Lee et al. 2014; Liu and Shell 2012) generally take an approach that considers only a fixed set of tasks, equivalent to a “one size fits all” architecture, limiting the ability of a system to adapt to different hardware configurations, and reducing the opportunities for optimisation.

Instead, we advocate the development of systems based on the selection and allocation of what we term “task variants”. Task variants are interchangeable software components that offer configurable levels of quality of service (QoS) with a corresponding difference in the amount and/or type of computing resources they demand; such variants naturally arise in many scenarios, and often deployed systems consist of a particular subset of variants that have been implicitly chosen by a system architect. For example, consider alternative feature detection algorithms to solve a common task in a robotics vision pipeline: different algorithms provide increasingly sophisticated recognition methods but at the cost of increasing CPU load. Similarly, a variant may offer accelerated processing by targeting specialised hardware (e.g. GPUs, FPGAs).

Currently, the crucial step of selecting and allocating such task variants is typically performed using ad-hoc methods, which provide no guarantee of optimality and may thus lead to inefficient allocation. In this paper, we take a more systematic approach. We formalise the task variant allocation problem and propose three different solution methods that are able to efficiently exploit the available resources with the objective of maximising QoS while ensuring system correctness.

**Fig. 1:**
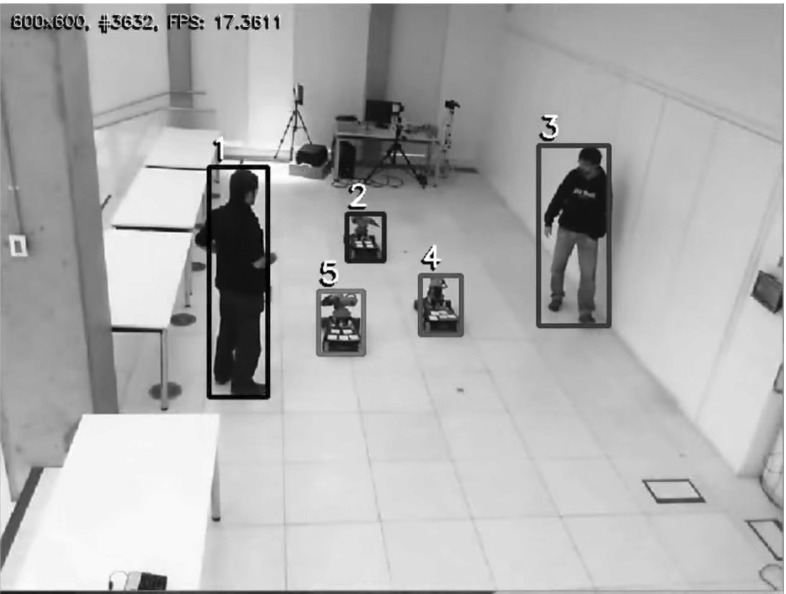
Case study: multi-agent navigation system composed of autonomous robots (KUKA youBots), humans, and network cameras

We focus on distributed heterogeneous robotics systems where variants are naturally available for several tasks. In particular, our work has been driven by a case study (Fig. 1), in the form of a distributed system of agents running on ROS (Quigley et al. 2009). The application implements a framework for inferring and planning with respect to the movement of goal-oriented agents in an interactive multi-agent setup—full details can be found in Bordallo et al. (2015). There are two types of agents navigating in the same physical space: autonomous robots represented by KUKA youBots (Bischoff et al. 2011) and humans. Each agent is pursuing a goal (a specific spatial position in the scenario) while avoiding collisions with other agents, based on online sensor processing and beliefs concerning the latent goals of other agents.

Specific tasks are used to accomplish this objective in a distributed fashion. For example, robots infer navigation goals of other agents from network camera feeds, provided by at least one *Tracker* task—meanwhile humans act independently and are assumed to navigate as a rational goal-oriented agent through the space. Some tasks can be configured via parameter values (e.g. the camera frame rate for the *Tracker* task) that translate into variants for that task. Each of these variants produces a different level of QoS, which we assume is quantified by an expert system user. Thus, the objective is to select task variants and allocate them to processors so as to maximise the overall QoS while agents reach their goals.

The contributions of the paper are as follows: (i) we introduce a mathematical model that represents the task variant selection and allocation problem; (ii) we propose three different solution methods (constraint programming, local search metaheuristic, greedy heuristic) to the problem; (iii) we evaluate and compare the solution methods through simulation; (iv) we validate the solution methods in a real-world interactive multi-agent navigation system, showing how our best solution method (constraint programming) clearly outperforms the average QoS of the local search metaheuristic by 16%, the greedy heuristic by 31%, and a random allocation by 56%. To the best of our knowledge, we are the first to address task allocation in the presence of variants in distributed robotics.

## Problem formulation

We now model the problem of task variant allocation in distributed robotics, in a general formulation that also applies to the specifics of our case study. We consider allocation as a constrained type of multi-objective, multi-dimensional, multiple-choice knapsack problem. Whilst instances of these three problems are individually common in the literature (Kellerer et al. 2004; Martello and Toth 1990), the combination is not. In addition, we allow for a number of unusual constraints describing task variants that distinguish this formulation from previous work (e.g. the specific type of hardware required to run a variant). Note that in this work we consider task allocation from a static point of view, although a dynamic case could be addressed as a process of repeated static allocations, or a more sophisticated method could be developed. We leave the dynamic case for future work.

Our formulation of the problem divides cleanly into three parts: the *software* architecture of the system, including information about task variants; the *hardware* configuration that is being targeted as a deployment platform; and the constraints and goals of task *selection and allocation*, which may be augmented by a system architect.

### Software model

A software architecture is defined by a directed graph of tasks, (*T*, *M*) where the set of tasks $T=\{\tau _1\ldots \tau _n\}$ and each task $\tau _i$ is a unit of abstract functionality that must be performed by the system. Tasks communicate through message-passing: edges $m_{i,j}~=~(\tau _i,~\tau _j)~\in ~M \subseteq T \times T$ are weighted by the ‘size’ of the corresponding message type, defined by a function $S:\ m_{i,j} \rightarrow \mathbb {N}$; this is an abstract measure of the bandwidth required between two tasks to communicate effectively.

Tasks are fulfilled by one or more *task variants*. Each task must have at least one variant. Different variants of the same task reflect different trade-offs between resource requirements and the QoS provided. Thus a task $\tau _i$ is denoted as set of indexed variants: $\tau _i = \{v_1^i\ldots v_n^i\}, \tau _i \ne \emptyset$. For convenience, we define $V = \cup _{i}\ \tau _i$, such that *V* is the set of all variants across all tasks. For simplicity, we make the conservative assumption that the maximum message size for a task $\tau _i$ is the same across all variants $v_j^i$ of that task, and we use this maximum value when calculating bandwidth usage for any task variant. Note that this assumption could only impact the overall solution in highly constrained networks, which is very unlikely nowadays. For example, in our case study the maximum data rates required are very low (45 KB/s) compared to the available bandwidth (300 MB/s).

A given task variant $v_j^i$ is characterised by its processor utilisation and the QoS it provides, represented by the functions $U, Q: v_j^i \rightarrow \mathbb {N}$. The utilisation of all task variants is expressed normalised to a ‘standard’ processor; the capacity of all processors is similarly expressed. QoS values can be manually (Sect. 5.1) or automatically generated (future work), although this is orthogonal to the problem addressed.

### Hardware model

The deployment hardware for a specific system is modelled as an undirected graph of processors, (*P*,  *L*) where the set of processors $P~=~\{p_1\ldots p_n\}$ and each processor $p_k$ has a given processing capacity defined by a function $D : p_k \rightarrow \mathbb {N}$. A bidirectional network link between two processors $p_k$ and $p_m$ is defined as $l_{k,m} = (p_k, p_m) \in L~\subseteq ~P~\times ~P$, so that each link between processors will support one or more message-passing edges between tasks. The capacity of a link is given by its maximum bandwidth and is defined by a function $B:\ l_{k,m} \rightarrow \mathbb {N}$. If in a particular system instance multiple processors share a single network link, we rely on the system architect responsible for specifying the problem to partition network resources between processors, such as simply dividing it equally between processor pairs.

### Selection and allocation problem

The problem hence is to find a partial function $A : V \rightarrow P$, that is, an assignment of task variants to processors that satisfies the system constraints (i.e. a *feasible* solution), whilst maximising the QoS across all tasks, and also maximising efficiency (i.e. minimising the average processor utilisation) across all processors. As *A* is a partial function, we must check for domain membership of each task variant, represented as *dom*(*A*), to determine which variants are allocated.

We assume that if a processor is not overloaded then each task running on the processor is able to complete its function in a timely manner, hence we defer the detailed scheduling policy to the designer of a particular system.

An optimal allocation of task variants, $A^*$, must maximise the average QoS across all tasks (i.e. the global QoS):

1 $$\begin{aligned} max \quad 1/|T| \sum \limits _{v_j^i \in dom(A)} Q(v^i_j) \end{aligned}$$

Whilst minimising the average utilisation across all processors *as a secondary goal*:

2 $$\begin{aligned} min 1/|P| \sum _{p_k \in P} \sum \limits _{ v_j^i \in dom(A): ~A( v_j^i ) = p_k } U(v_j^i) \end{aligned}$$

Exactly one variant of each task must be allocated:

3 $$\begin{aligned}&\forall \tau _i \in T,~\forall v_j^i, v_k^i \in \tau _i : \nonumber \\&\quad (v_j^i \in dom(A) \wedge v_k^i \in dom(A)) \implies j = k \end{aligned}$$

The capacity of any processor must not be exceeded:

4 $$\begin{aligned} \forall p_k \in P : \big (\sum \limits _{ v_j^i \in dom(A): ~A( v_j^i ) = p_k } U(v_j^i) \big ) \le D(p_k) \end{aligned}$$

The bandwidth of any network link must not be exceeded:

5 $$\begin{aligned} \forall l_{q,r} \in L : \Big ( \sum \limits _{i : A( v_j^i ) = p_q } \sum \limits _{k : A(v_l^k)=p_r} S(m_{i,k}) + S(m_{k,i}) \Big ) \le B(l_{q,r}) \end{aligned}$$

In addition, *residence constraints* restrict the particular processors to which a given task variant $v_j^i$ may be allocated, to a subset $R^i_j~\subseteq ~P$. This is desirable, for example, when requisite sensors are located on a given robot, or because specialised hardware such as a GPU is used by the variant:

6 $$\begin{aligned} v_j^i \in dom(A) \implies ~A(v_j^i) = p_k \in R^i_j \end{aligned}$$

*Coresidence constraints* limit any assignment such that the selected variants for two given tasks must always reside on the same processor. In practice, this may be because the latency of a network connection is not tolerable. The set of coresidence constraints is a set of pairs $(\tau _i, \tau _k)$ for which:

7 $$\begin{aligned}&\forall v_j^i \in \tau _i, \forall v_l^k \in \tau _k:~(v_j^i \in dom(A)~\wedge ~v_l^k \in dom(A))\nonumber \\&\quad \implies A(v_j^i) = A(v_l^k) \end{aligned}$$

## Solution methods

We now propose and describe our three different centralised approaches to solving the problem of task variant allocation^1^: constraint programming (CP), a greedy heuristic (GH), and local search metaheuristic (LS). These are three broadly representative search techniques from diverse families of solution methods, as outlined by Gulwani (2010).

### Constraint programming

We expressed the problem in MiniZinc 2.0.11 (Nethercote et al. 2007), a declarative optimisation modelling language for constraint programming. A MiniZinc model is described in terms of variables, constraints, and an objective. Our model has a variable for each variant, stating the processor it is to be assigned to; since we are constructing a partial mapping, we add a special processor to signify an unassigned variant. Matrices are used to represent the bandwidth of the network and the sizes of messages exchanged between tasks.

Most constraints are a direct translation of those in Sect. 2.3 although the constraint given by Eq. 3 is expressed by saying that the sum of the variants allocated to any given task is one—this natural mapping is why we selected MiniZinc, rather than (for example) encoding to mixed integer programming (Wolsey 2008). The development of a model that allows MiniZinc to search efficiently is key to its success, and we spent some time refining our approach to reduce solution time. However, the model could be further refined. For example, we could investigate non-default variable and value ordering heuristics, or introduce a custom propagator which avoids the $O(n^4)$ space associated with encoding the bandwidth constraints.

There are two objectives to be optimised, and we achieve this by implementing a two-pass method: first the QoS objective is maximised, we parse the results, and then MiniZinc is re-executed after encoding the found optimal value as a hard constraint whilst attempting to minimise processor utilisation—note that the MiniZinc model doesn’t include the 1 / |*P*| and 1 / |*T*| terms in the objective, since floats or divisions affect constraint programming performance considerably. Instead, we apply these terms in the Python program that calls MiniZinc.

The full model is available online (White and Cano 2017), but to give a flavour, we show our variables, a constraint, and the first objective:
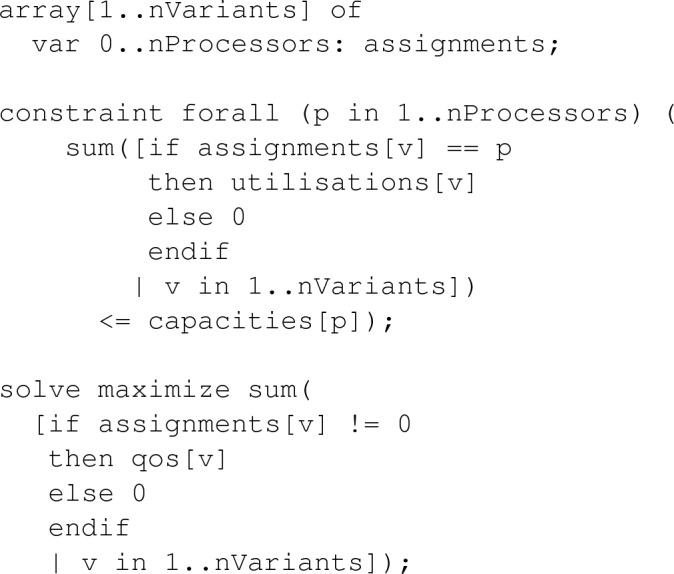


MiniZinc allows instance data to be separated from the model. Part of a data file looks like this:
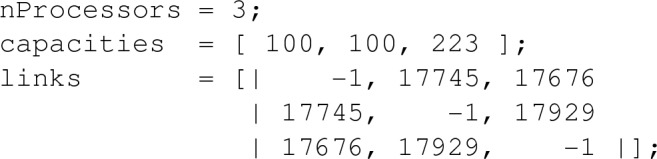


To solve instances, we used the *Gecode* (Gecode Team 2006) constraint programming toolkit, which combines backtracking search with specialised inference algorithms. We used the default search rules, and only employ standard toolkit constraints. It addition to being used as an exact solver, *Gecode* can also run in *anytime* fashion, such that it reports the best solution found so far. Our system reports its progress in terms of the best-known solution at any point during the execution of the solver, as well as the optimal result, where found. In our evaluation we consider both the standard mode, which returns the global optimum after an unrestricted runtime (Sect. 5.3), and also this anytime mode that returns the best result found so far (Sect. 5.5).

### Greedy heuristic

Our second solution method is a non-exact greedy algorithm that uses a heuristic developed from an algorithm originally designed for solving a much simpler allocation problem (Cano et al. 2015). The procedure is described in Algorithm 1, and attempts to obey constraints, then allocate the most CPU intensive tasks possible to those processors with the greatest capacity.
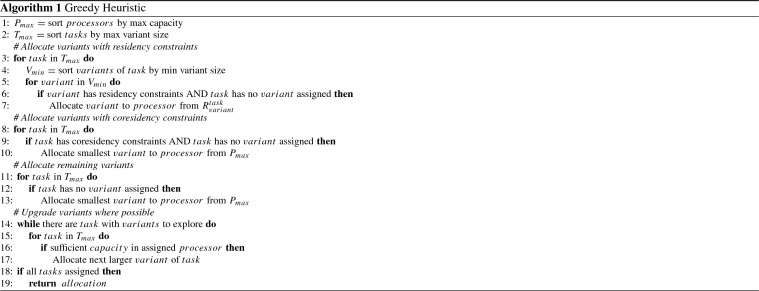
 
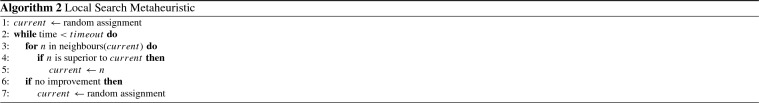


First, the smallest task variants with residency constraints are allocated to processors (lines 3–7), beginning with the largest capacity processor if the subset $R^i_j$ for a given task variant $v^i_j$ contains more than one element. Next, the smallest variants of any tasks with coresidency constraints are assigned selecting processors from $P_{max}$ (lines 8–10). Then, the smallest variants of any remaining, unallocated, tasks are allocated, again preferring processors with more capacity (lines 11–13). Finally, the algorithm iteratively attempts to substitute smaller variants with larger ones on the same processor (lines 14–17). Note that the way in which the next processor (from $R^i_j$, $P_{max}$) or variant is selected must also ensure that allocations will not result in a violation of any previously satisfied constraints.

The greedy heuristic is not guaranteed to find a solution, but if it finds one it is always feasible, i.e. satisfies the system constraints. The ability to provide solutions is greatly determined by residency and coresidency constraints.

### Local search metaheuristic

The third algorithm we propose is a simple local search metaheuristic employing random restarts. The process is described by Algorithm 2. Initially, a random assignment is generated by allocating a random variant for each task to a random processor (line 1), and all choices are made uniformly random. There is no guarantee a randomly generated allocation will satisfy the constraints of the model, and indeed the search algorithm is not guaranteed to find a feasible solution. Neighbouring solutions are generated (line 3) and accepted if the resulting allocation is superior to the incumbent one (lines 4–5). As there is no way to determine if the global optimum has been found, the algorithm continues to search the space of assignments until a given timeout is reached. The search may find a local optimum, in which case a random restart is used to explore other parts of the search space (lines 6–7).

The neighbourhood of a solution in the space of allocations is defined as all those solutions that can be generated by substituting another variant of the same task for one already allocated, or alternatively by moving a single variant to a different processor. In order to determine if one solution is preferable to another, a priority ordering amongst the constraints and objectives is established, in order of importance:

1. No processors should be overloaded.
2. The network should not be overloaded.
3. Residency constraints must be satisfied.
4. Coresidency constraints must be satisfied.
5. Average QoS per task should be maximised.
6. Average free capacity per CPU should be maximised.

A solution is feasible if the first four constraints are satisfied, after which the search will try to optimise QoS and then reduce processor utilisation to free up capacity. This priority ordering method is preferred over the alternative of a *weighted sum objective*, an approach found elsewhere in the literature (Marler and Arora 2009). Weighted sum approaches require the user to precisely quantify the relative importance of objectives and constraints, which is a somewhat inelegant approach to this problem, as it can be unrealistic in many scenarios (e.g. when considering factors such as execution time, energy consumption, and functional performance). Furthermore, it is known that some members of the pareto front will not be found when using such an approach (Coello et al. 2006; Das and Dennis 1997). As we prioritise functional performance over non-functional concerns, a two-stage approach is more appropriate. For the same reason, we prefer local search over *simulated annealing* (Tindell et al. 1992), an algorithm we also experimented with, which relies on a numerical gradient in the constrained objective space as a measure of absolute quality (i.e. it requires to provide weightings in the same manner as a weighted sum approach, which suffers from the problems given above).

### Computational complexity

In the worst case, hill-climbing has time complexity $O(\infty )$, i.e. it may never find the global optimum. Our implementation of hill-climbing (i.e. local search metaheuristic) uses the *random restart* strategy, that is, it commences a new search once it has found a local optima, but this still does not guarantee it will find the global optimum. Similarly, greedy-search cannot provide such a guarantee. Finally, the worst-case complexity of the constraint programming approach is in principle exponential, but our results show that this does not occur in practice on the datasets analysed.

## Example case study

Our case study serves as a specific instantiation of the general formulation presented, with which we can test our algorithmic solutions in a real system. We first present a *baseline instance* of the system, consisting of a single robot, person, server and camera. This simplified configuration illustrates the system components and the constraints imposed on them. Each robot or human agent is pursuing a spatial goal. The application’s overarching QoS metric is a combination of essential requirements (e.g. avoid collisions between agents, minimise travel time to reach target goals), as well as more sophisticated preferences (e.g. minimise close-encounters and hindrance between navigating agents, minimise the time taken to infer the true agent goal). Therefore, task variants must be selected and allocated across available processors with the objective of optimising global QoS based on the selected variants’ individual QoS values.

### Software architecture

**Fig. 2:**
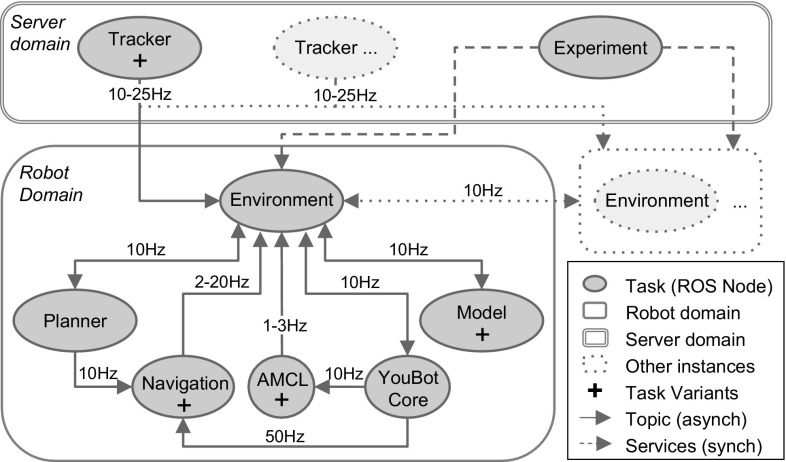
Case study software architecture, composed of one *Tracker* instance per camera, one instance of each task in the *Robot domain* per robot, and one *Experiment* instance for the complete system

Figure 2 shows a high-level diagram representing the software architecture of the case study. It is composed of multiple tasks and their message connections. In the figure, connections are labelled with message frequencies, which can be obtained from the maximum bandwidth requirement described in Sect. 2.1. The QoS values for the variants of a given task represent the proportional benefit of running that task variant; a variant that has a higher QoS, however, would typically incur a higher CPU usage. We rely on an expert system user to estimate QoS values for task variants.

**Table 1:** Task variants characterisation

Task	Variants	Parameters	CPU	Freq (Hz)	BW (KB/s)	Res	CoRes	QoS
Experiment	1	–	1	10	1	Server	–	1
Tracker	4	Output freq. (25 20 15 10)	200 160 120 80	25 20 15 10	2.5 2 1.5 1	Server	–	100 90 70 40
Environment	1	–	1	10	0.5	–	–	1
Model	3	Num. goals (10000 3500 4)	59 39 17	10 10 10	5 5 5	–	–	100 60 20
Planner	1	–	1	10	0.5	–	Navig.	1
AMCL	3	Particles (3000 500 200)	66 41 19	2.5 2.5 2.5	1 1 1	–	–	100 75 50
Navigation	3	Controller freq. (20 10 2)	50 39 25	20 10 2	1 0.5 0.1	–	Planner	100 67 33
Youbot_Core	1	–	16	10	0.5	Robot	–	1

We now describe for each task in our case study, the corresponding variants (see Table 1 for details):

- *Tracker* A component of a distributed person tracking algorithm that fuses multiple-camera beliefs using a particle filter. The variants for this task are based on the input image resolution and the output frame rate given a fixed number of cameras. The higher the output frame rate the more accurate the tracking.
- *Experiment* A small synchronous task that coordinates all robots taking part in the experiment.
- *Environment* A local processing task required by each robot. This task combines information generated by the local robot, other robots, and elsewhere in the system (i.e. *Tracker*, *Experiment*).
- *Model* An intention-aware model for predicting the future motion of interactively navigating agents, both robots and humans. The variants for this task are based on the number of hypothetical goals considered given a fixed number of agents. A higher number of modelled agent goals will lead to more accurate goal estimates.
- *Adaptive Monte Carlo Localisation (AMCL)* Localisation relying on laser data and a map of the environment (Pfaff et al. 2006). The variants of this task vary with the number of particles used during navigation, since a larger number increases localisation robustness and accuracy in environments populated with other moving obstacles. We assume the robot moves on average at the preferred speed of 0.3 m/s (min 0.1 m/s, max 0.6 m/s).
- *Planner* Generates an interactive costmap, which predicts the future motion of all agents with relation to other agents’ motion given their inferred target goals. Since the costmap is used by the *Navigation* task for calculating the trajectory to be executed, the two tasks have a coresidence constraint to guarantee a proper behaviour.
- *Navigation* This task avoids detected obstacles and attempts to plan a path given the interactive costmap of the agents in the environment, ultimately producing the output velocity the robot platform must take. The variants of *Navigation* depend on the controller frequency, that is, the number of times per second the task produces a velocity command. The higher the frequency, the more reactive and smooth the robot navigation becomes.
- *YouBot_Core* A set of ROS packages and nodes that enable the robot to function, for example *etherCAT* motor connectivity, internal kinematic transformations, and a laser scanner sensor. This task must always run in the corresponding robot (i.e. it has a residence constraint).

Finally, we assume that a robot is capable of executing a full set of its tasks, at the very least by selecting their least-demanding variants. Those tasks are represented within the robot domain in Fig. 2. This is critical to ensure a continued service in periods of network outage in future dynamic scenarios, albeit at lower levels of QoS.

### Hardware architecture

The hardware integrating the baseline system is composed of a single network camera and two processors, that is, a robot with onboard processor and a remote server. Robot and server communicate through a wireless network, and camera and server through a wired network. In practice the network bandwidth is currently not a limiting factor, as both networks are dedicated and private in our lab. The same applies to the latency/quality of the wireless signals.

## Evaluation

In this section, we first describe the results of an empirical characterisation of the baseline system, which is mandatory to evaluate both the solution methods and the case study itself. We then extend this characterisation to define a set of system instances of increasing size and complexity. Having established these benchmark problems, we employ them to evaluate the utility of our solution methods, in two stages.

In the first stage, we compare the quality of solutions returned by the three proposed methods to answer the following research questions:

- *RQ1A* Is it possible to find globally optimal variant selections and allocations using constraint programming?
- *RQ1B* How well can a straightforward greedy heuristic and the local search metaheuristic perform on this problem, relative to the constraint programming method?
- *RQ1C* How does the solution time scale with the size and complexity of the example system instances?
- *RQ1D* How well do the results produced by the three solution methods translate to deployment on the physical system outlined in Sect. 4?
- *RQ1E* How effective are the allocations proposed by our solution methods compared to random allocations?

In the second stage, we compare the constraint programming solver configured in *anytime* mode against the local search metaheuristic, to explore their performance over time. Our research questions are as follows:

- *RQ2A* How do local search metaheuristic and “anytime” constraint programming compare in terms of their solutions quality after a given period of run-time?
- *RQ2B* Could these two “anytime” methods be used in future dynamic scenarios?

### System characterisation

We performed an offline characterisation of the baseline system shown in Fig. 2 using common monitoring utilities from ROS (e.g. *rqt*, which provides average values) and Linux (e.g. *htop*, visually inspecting it during execution). The objective was to obtain for each unique task variant in the system the following values: (i) the average percentage of CPU utilisation required; (ii) the average frequency at which messages published are sent to other tasks; and (iii) the average network bandwidth required.

Table 1 summarises the values obtained. Column two represents the number of variants for each task, and column three the value of the parameters that create the task variants (see Sect. 4.1). The next three columns include the average values of CPU utilisation, frequency and bandwidth for each task variant—note that the maximum values for frequency are shown in Fig. 2. The CPU values for the *Tracker* task assume only one person in the environment. Columns seven and eight show the residence and coresidence constraints for each variant and task respectively. Finally, the last column represents the normalised QoS associated with each task variant, where 100 is the maximum value. Note that we have assigned QoS value “1” to single variant tasks because they have much less impact in the system behaviour, which is reflected in low CPU utilisation values in Table 1. The focus of this work is task variant allocation, for which we require QoS values as inputs. Although QoS values were manually generated based on real system measurements, they may be automatically generated, but we leave this for future work. It is worth noting that the user is required to provide QoS values only “once” for each task variant. Therefore, when the system is scaled up by replicating tasks on more robots or cameras, no further manual characterisation work is required from the user.

Finally, we specify the hardware characteristics of the baseline system. The robot’s on-board processor is an Intel Atom, 2 cores @ 1.6 GHz, 2 GB RAM. The server’s processor is an Intel i5-3340, Quad Core @ 3.30 GHz (Turbo), 16 GB RAM. Note that all CPU measurements are normalised to the robot CPU capacity (assumed as 100). From this, we can understand why the *Tracker* instances (which have a high CPU requirement) can only run in the server, translating into a residence constraint. The networks employed are a wireless IEEE 802.11ac network at 300 Mbps, and a wired 1 Gbps Ethernet network.

### System instances

In order to obtain more complex instances of the baseline system shown in Fig. 2, we only need to add processors (i.e. robots, servers) and/or cameras, allowing the system to cope with a more complex environment and complete more difficult challenges. As these parameters are varied, the total number of tasks and variants changes accordingly, but the number of variants for each task is fixed.

**Table 2:** System instances considered

Inst.	Proc.	Robots	Cam	Tasks	Var.	S. space $N_k$
1	2	1	1	8	17	1728
2	2	1	2	9	21	6912
3	2	1	3	10	25	27,648
4	3	2	1	14	29	$1.91 \times 10^{5}$
5	3	2	2	15	33	$7.65 \times 10^{5}$
6	3	2	3	16	37	$3.06 \times 10^{8}$
7	4	3	1	20	41	$1.32 \times 10^{12}$
8	4	3	2	21	45	$5.28 \times 10^{12}$
9	4	3	3	22	49	$2.11 \times 10^{13}$
10	4	3	4	23	53	$8.45 \times 10^{13}$
11	5	4	1	26	53	$4.45 \times 10^{14}$
12	5	4	2	27	57	$1.78 \times 10^{15}$
13	5	4	3	28	61	$7.12 \times 10^{15}$
14	5	4	4	29	65	$2.85 \times 10^{16}$

Table 2 summarises the set of instances comprising our benchmarks, which includes the number of tasks and variants generated for each case—note that only one server with a capacity of 400 is used for all cases. In order to provide an estimate of the search space size, an approximate upper bound $N_k$ for the number of possible variant assignments for a given problem instance *k* is calculated as follows:

8 $$\begin{aligned} N_k = \prod \limits _{\tau _i\ \in \ T} | P(\tau _i)| \cdot | \tau _i | \end{aligned}$$

where $|P(\tau _i)|$ is the number of possible processors on which a task $\tau _i$ can be allocated without violating residency and coresidency constraints, and $| \tau _i |$ is the size of the set of variants of the task. The size of the search space $N_k$ for the instances considered is shown in the last column of Table 2.

**Table 3:** Total execution time of greedy heuristic (GH), local search (LS), and constraint programming (CP) for the system instances considered

Instance	1	2	3	4	5	6	7	8	9	10	11	12	13	14
GH	3 ms	5 ms	6 ms	10 ms	13 ms	14 ms	21 ms	23 ms	22 ms	25 ms	30 ms	29 ms	33 ms	37 ms
CP/LS	340 ms	340 ms	390 ms	1.36 s	2.34 s	3.54 s	14.20 s	9.6 m	33 m	17 m	10.1 h	6.19 d	2 w	6.81 d

*ms* milliseconds, *s* seconds, *m* minutes, *h* hours, *d* days, *w* weeks

### Simulation results

We now analyse and compare the QoS and CPU utilisation values of solutions provided by the three proposed methods (since these are simulation results,^2^ we call them expected values). Remember that the allocation of more powerful variants translates into higher global QoS values, and strongly correlates with improved overall system behaviour. For example, switching from the least to most powerful variant of the *Tracker* task (QoS values 40 and 100 in Table 1) actually provides more accurate and faster tracking of people in the environment. This in turn provides the *Planner* and *Model* tasks with better data, improving the robots ability to navigate (e.g. avoiding collisions).

**Fig. 3:**
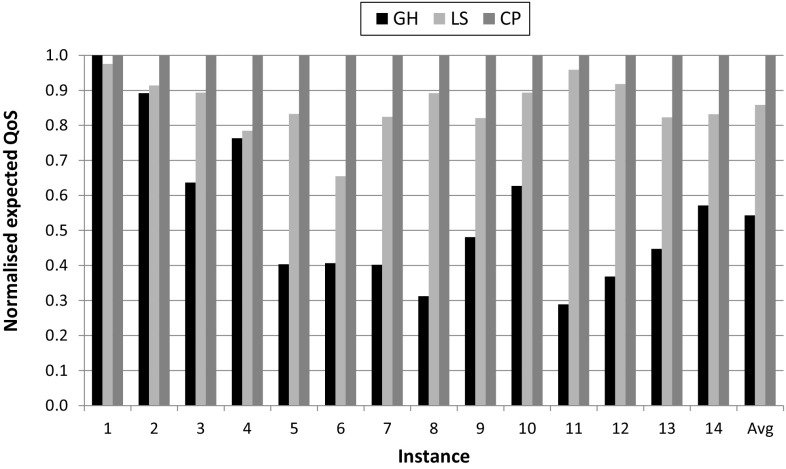
Expected QoS for greedy heuristic (GH), local search (LS), and constraint programming (CP). Values are normalised to the optimal solution (= 1). Server capacity = 400

We execute Python programs implementing the three proposed methods for the instances described in Table 2. All simulation experiments were performed on a 2.8 GHz Intel Core i7 with 4 GB RAM (Table 3 shows the total execution times). Answering *RQ1A*, we found that constraint programming finds the globally optimal solution for all instances analysed. In other words, for each instance this method provides the allocation of task variants to processors with the best possible average QoS and minimum CPU utilisation. Since constraint programming provides the best possible QoS, we normalise the QoS provided by the greedy and local search methods to the optimum. Figure 3 shows results comparing the QoS of the three methods—note that values for local search are actually the average of three independent runs with a timeout set to be equal to the time required by the constraint programming method. Therefore, answering *RQ1B*, we observe that local search and greedy heuristic achieve an average of 14 and 46% lesser QoS than constraint programming respectively.

**Fig. 4:**
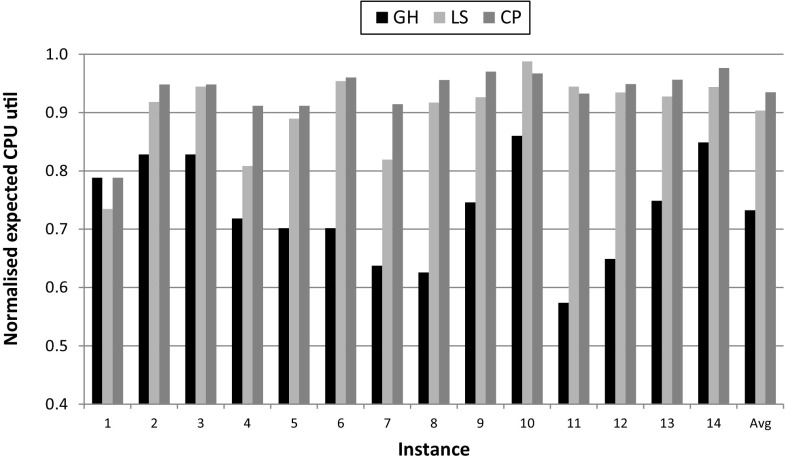
Expected CPU utilisation for greedy heuristic (GH), local search (LS), and constraint programming (CP). Values are normalised to the corresponding total CPU capacity (= 1). Server capacity = 400

Figure 4 shows the results for CPU utilisation, where the values indicated refer to the total utilisation of the sum of all CPU capacities. On average, constraint programming utilises 3 and 20% more CPU capacity than local search and greedy respectively, but as shown in Fig. 3, the differences in QoS are much greater (14% and 46%). There are two special cases in Fig. 4 where local search utilises more CPU capacity than constraint programming while providing lesser QoS. For $Instance\ 10$, the reason is that local search finds an infeasible solution. However for $Instance\ 11$ the solution found is feasible, which further demonstrates that constraint programming provides better solutions—recall that it uses a two-pass method.

Since we maintain the server capacity (= 400) across all instances analysed, the problem becomes more constrained as the total number of task variants increases. As an example, constraint programming and the greedy heuristic solve $Instance\ 1$ allocating the most powerful variant for all tasks ($V_{CP} = 1$ and $V_{GH} = 1$ in Table 4). Note how constraint programming balances much better the allocation of task across the available processors ($P_{CP}$ and $P_{GH}$ in Table 4). However for $Instance\ 10$ (Table 5), some tasks need to use less powerful variants in order to satisfy the CPU capacity constraint (e.g. the four *Tracker* tasks use the least powerful variant for constraint programming, $V_{CP} = 4$). In Table 5 we also see how local search allocates $Tracker\_1$ to $Processor\ 3$ ($P_{LS} = 3$), thus providing the infeasible solution commented previously. Since the *Tracker* task has a residence constraint, it can only be allocated to the server, which is $Processor\ 4$ for this instance.

**Table 4:** $Instance\ 1$ (1 robot, 1 camera): task variant selection (V is the variant, smaller numbers indicate more powerful) and allocation (P is the processor, biggest number is the server) for each solution method

Tasks (8)	$V_{GH}$	$P_{GH}$	$V_{LS}$	$P_{LS}$	$V_{CP}$	$P_{CP}$
Experiment	1	2	1	2	1	2
Tracker	1	2	3	2	1	2
Environment	1	2	1	2	1	1
Model	1	2	1	1	1	2
Planner	1	2	1	2	1	1
AMCL	1	2	1	2	1	2
Navigation	1	2	1	2	1	1
Youbot_Core	1	1	1	1	1	1

**Table 5:** $Instance\ 10$ (3 robots, 4 cameras): task variant selection (V is the variant, smaller numbers indicate more powerful) and allocation (P is the processor, biggest number is the server) for each solution method

Tasks (23)	$V_{GH}$	$P_{GH}$	$V_{LS}$	$P_{LS}$	$V_{CP}$	$P_{CP}$
Experiment	1	4	1	4	1	4
Tracker_1	4	4	4	**3**	4	4
Tracker_2	4	4	3	4	4	4
Tracker_3	4	4	4	4	4	4
Tracker_4	4	4	4	4	4	4
Environment_1	1	2	1	3	1	1
Model_1	2	3	3	3	2	3
Planner_1	1	4	1	1	1	2
AMCL_1	3	2	2	1	3	3
Navigation_1	3	4	2	4	1	2
Youbot_Core_1	1	1	1	1	1	1
Environment_2	1	4	1	2	1	1
Model_2	3	2	1	4	2	4
Planner_2	1	4	1	2	1	1
AMCL_2	3	2	1	2	3	2
Navigation _2	3	4	3	4	2	1
Youbot_Core_2	1	2	1	2	1	2
Environment_3	1	2	1	3	1	1
Model_3	2	3	3	1	2	4
Planner_3	1	4	1	3	1	1
AMCL_3	3	2	3	4	3	3
Navigation_3	3	4	3	1	2	1
Youbot_Core_3	1	3	1	2	1	3

Finally, and answering *RQ1C*, we see that the solution times for constraint programming are reasonable up to $Instance\ 10$ (Table 3), which is actually a big system in terms of the search space (Table 2). At this point, the times start to become intractable (we analyse the anytime behaviour in Sect. 5.5). Thus, we don’t report results for larger system instances. We leave further refinements to our MiniZinc model as future work, which could potentially allow scaling to larger instances. On the other hand, the solution times for the greedy heuristic are small (milliseconds) and scale well, although it provides inferior QoS values to constraint programming, as previously discussed.

### Analysis of case study behaviour

Having obtained the simulation results, our next step is to validate that the expected QoS values obtained via simulation match the behaviour of the real system. To do this, we performed experiments for instances 1–6 from Table 2 in our case study environment. For each instance, we configured the allocation of task variants to processors computed by the solution methods—note that only a single human agent is present in the environment for all experiments. Then, the measured QoS value for each instance and method is obtained by applying the following formula:

9 $$\begin{aligned} QoS_{ measured } = \sum _{\tau \in T} QoS_{\tau }\times \frac{F^o_{\tau }}{F^e_{\tau }} \end{aligned}$$

where $QoS_{\tau }$ is the expected QoS value for task $\tau$ as predicted by our solution methods, ${F^o_{\tau }}$ is the observed frequency of messages produced by task $\tau$ on the real system and ${F^e_{\tau }}$ is the expected frequency associated with task $\tau$ (Table 1). These two frequencies can differ due to overloaded processors (for infeasible solutions) and/or approximation errors in the system characterisation. Therefore, this frequency ratio determines the effectiveness of a task variant in the real system.

**Fig. 5:**
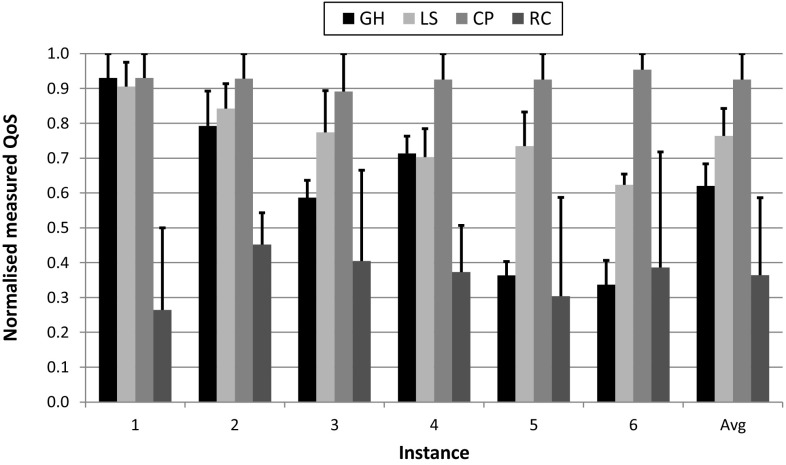
Validation of simulation results on the physical system: measured QoS for greedy heuristic (GH), local search (LS), constraint programming (CP), and random allocations (RA). Error bars represent the deviation from the expected QoS values

Figure 5 shows the results. The black error bar for each column denotes the difference between measured QoS (top of column) obtained with Eq. 9, and expected QoS (error bar upper end) obtained by simulation. Answering *RQ1D*, the measured QoS values for local search, constraint programming and the greedy heuristic only deviate by 8, 7 and 5% on average respectively from the expected values. This result validates the accuracy of our methodology.

Finally, we also examined the system behaviour considering random allocations of task variants to processors—note that this could be the best choice for users with little knowledge of the system or for large system instances. Figure 5 also includes these results (RA), where each bar actually corresponds to the average QoS of three randomly generated allocations. Answering *RQ1E*, we see how the measured QoS values for random allocations deviate much more from the expected ones, by an average of 22%, than those for the proposed solution methods. The reason is that our solution methods produced feasible allocations for the six instances analysed (i.e. satisfying system constraints), thus differences are only due to approximation errors in the system characterisation. However, some of the random allocations produced infeasible solutions, which translated into overloaded processors and therefore larger differences with the expected values.

In summary, constraint programming improves the overall QoS of the real system by 16, 31, and 56% on average over local search metaheuristic, greedy heuristic and random allocations respectively.

### Anytime approaches

In this section, we consider the task allocation problem from a different point of view. If we are to apply our approach to larger systems, or select and allocate task variants at system boot, or even at run-time, then the time taken to find a good allocation takes on greater precedence. Both the *Gecode* constraint programming solver and local search solution methods can be used as *anytime algorithms*, where the best allocation currently known can be returned at any point during their execution. The two algorithms approach the problem differently, because constraint programming requires a two-pass procedure where each objective is optimised in turn, whereas the local search metaheuristic attempts to optimise both objectives simultaneously. Therefore, the relative performance of the two algorithms is of interest.

**Fig. 6:**
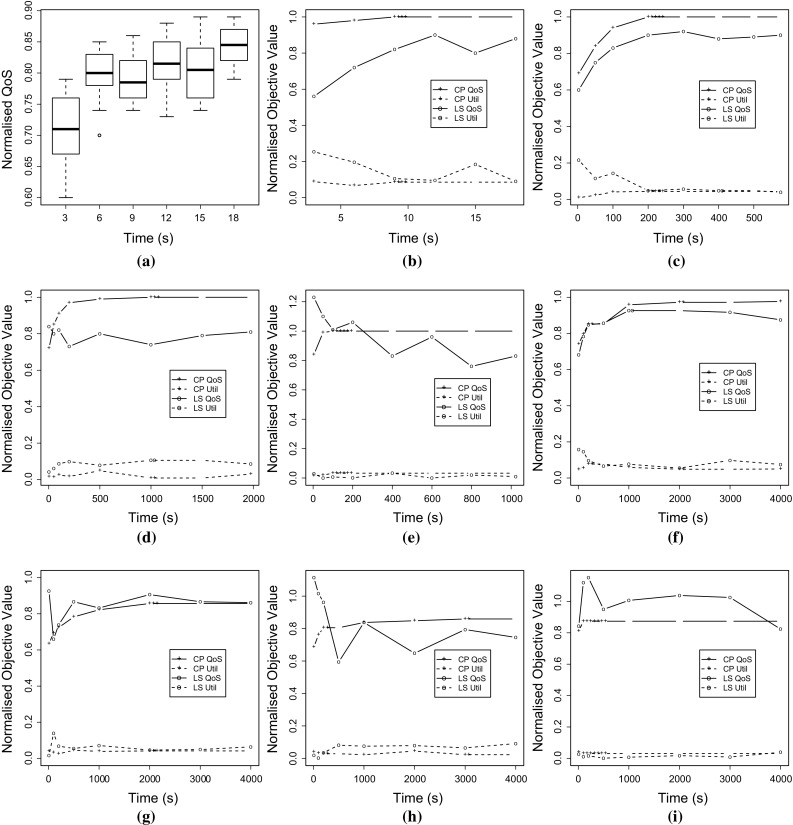
Anytime results for constraint programming (CP) and local search (LS): **a** Distribution on $Instance\ 7$ with 10 Repetitions; **b** $Instance\ 7$; **c** $Instance\ 8$; **d** $Instance\ 9$; **e** $Instance\ 10$; **f** $Instance\ 11$; **g** $Instance\ 12$; **h** $Instance\ 13$; **i** $Instance\ 14$

For our *anytime* analysis, we selected the instances that resulted in significant runtimes for constraint programming (Table 3), that is, from $Instance\ 7$ to $Instance\ 14$. Then, we executed the *Gecode* solver and the local search method for these instances with increasing timeout values, to evaluate how the solutions they found improved over time. Figure 6b–i show the results. The graphs show two objectives: firstly, the *Quality of Service* objective as defined by Eq. 1, and secondly the *Utilisation* objective as defined by Eq. 2. Each intermediary result is from an independent run of the algorithms, avoiding the problem of autocorrelation. All results in Fig. 6 are normalised to the optimal solution (“1"), which represents: (i) for QoS, the best possible value; (ii) for CPU utilisation, unutilised processors (free capacity of 100%). Note that for *Instances* 11 to 14 we only show the execution time for the first 4000 s, but in all cases the QoS obtained is greater than the 80% of the optimal value.

As can be observed for all the instances in Fig. 6, there is a certain amount of variance in the results produced by local search, based on the seed provided. For example, the fifth value for local search QoS in Fig. 6b is lower than the preceding and following values. To measure the variance for this instance, we repeated the experiment ten times, and present the results in Fig. 6a. This underlines the fact that the performance of local search is quite variable, although it generally makes steady progress over time.

The graphs in Fig. 6 illustrate a clear trend that answers *RQ2A*: in most cases, constraint programming produces superior results in the same amount of time, and is our preferred anytime solution method. The only exception in our results is $Instance\ 12$, where local search provides better QoS values for the last six points of the graph (Fig. 6g)—note that heuristic-based methods can randomly provide a good solution. For the first point ($time = 10$ s) local search provides an infeasible solution. The other instances containing points where local search is better than constraint programming (i.e. Fig. 6d, e, h, i) also correspond to infeasible solutions.

**Fig. 7:**
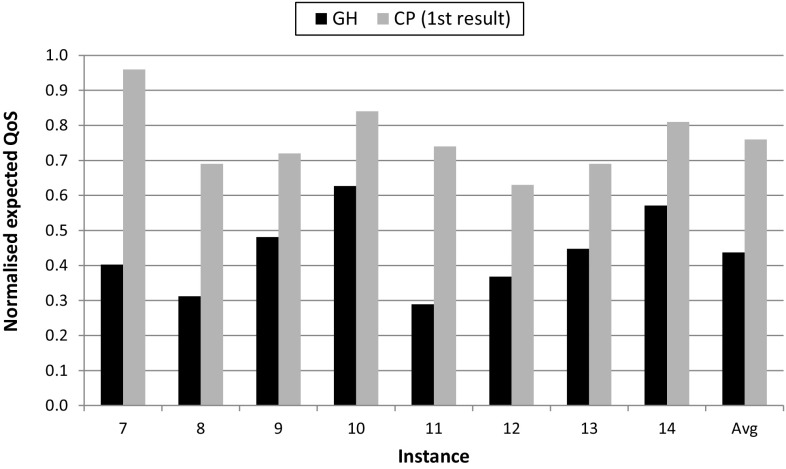
Expected QoS for for greedy heuristic (GH), and the first result provided by constraint programming (CP 1st result). Values are normalised to the optimal solution (=1). Server capacity = 400

Answering *RQ2B*, constraint programming also produces high quality results within a short timeframe, which may enable dynamic optimisation in the future and also increases our confidence in its ability to scale to larger systems. Figure 7 shows the first feasible solution provided by constraint programming normalised to the optimum. This first result is provided after 4 s for *Instances* 7–10, and after 10 s for *Instances* 11–14. The figure also shows the value for the greedy heuristic, just to have a clear idea of how good constraint programming is for these short times, a 32% better on average. Note that local search is not shown because after 10 s it provides infeasible solutions for almost all the instances.

Finally, note that since our local search algorithm is implemented in Python, it may be argued that constraint programming has an unfair advantage in that the MiniZinc solvers are written in C; however, the highly optimised nature of constraint solvers is actually a strong argument in favour of adopting them, particularly as they improve through continuous development over time.

## Related work

Much work has been performed in the area of task allocation in distributed robotics, where different types of optimisation problems have been addressed. A comprehensive taxonomy can be found in Korsah et al. (2013), where problems are categorised based on: (i) the degree of interdependence of agent-task utilities; and (ii) the system configuration, which in turn is based on an earlier taxonomy Gerkey and Matarić (2004) that considers the type of: agents, tasks and allocation. According to these taxonomies, the task variant allocation problem presented in this paper falls in the category of Cross-schedule Dependencies (XD), that is, the effective utility of each individual task-agent allocation depends on both the other tasks an agent is performing, and the tasks other agents are performing. Several types of system configurations are supported within this category—e.g. MT–SR–IA considers multi-task robots (MT), single-robot tasks (SR), and instantaneous task assignment (IA). Furthermore, problems in this category can be formulated with different types of mathematical models. In our case, we use a special form of knapsack formulation (Sect. 2).

Section 6.1 presents a systematic literature review of the area. Our key goals are to:

1. Quantify the size of typical search spaces for robot task allocation case studies.
2. Characterise the general solution methods used for task allocation in this domain.

This survey shows that our problem search spaces are larger than previous work, and our constraint programming optimisation technique is novel.

Section 6.2 proceeds to study key related work in distributed robotics falling in the same category as our work. We highlight how our work differs from past research.^3^ In systems like our case study, where task variants are instantiated by parameter configurations, it might be interesting to consider a greater number of variants for each task (even a continuum set), but then the allocation problem would require different solution methods, as shown in Cano et al. (2016a).

### Systematic review

This section presents a systematic review of the literature, following standard practice in the discipline (Kitchenham 2004). The research is performed to establish the search space boundaries in optimisation of task allocation for robotic applications. The survey was conducted so that we can quantitatively assess the maximum experiment size (either actual or simulated) of each published work included. This approach provides a comparative base between the scalability of existing work and our method in practice. In that respect we attempt a characterisation of limitations of task allocation approaches in the domain in comparison with the limitations of our approach.

Initially, three types of robotic systems were identified. The first type involved a larger number of robots performing less demanding tasks such as swarm systems. The second involved a moderate number of robots (10–30) (Sugiyama et al. 2016; Cheng et al. 2016). The third included a low number of robots performing very complex tasks (Khalfi et al. 2016; Wang et al. 2016; Hongli et al. 2016).

The strategy used for the systematic review involved searching for journal and conference publications through three well established databases and with a predefined combination of keywords. The databases are *IEEE Xplore* (IEEE 2016), *ScienceDirect* (Elsevier 2016) and *ACM Digital Library* (The Association for Computing Machinery 2016). Moreover, proceedings of well known conferences were reviewed and in particular *IROS, AAMAS, ICRA* and *RSS*. The search pattern used was *robot AND (“task allocation” OR “resource allocation”) AND (optimisation OR optimization)*. Moreover, to limit the search only the first 10 pages of results were examined for each of the databases. Further we restricted publications based on inclusion, exclusion and quality criteria below.

The inclusion criteria for published articles to be reviewed were:

1. Complex tasks.
2. Independently operating robots.
3. Task allocation either automated or manual.

The exclusion criteria were:

1. Simplistic tasks.
2. Small robots e.g. ant, swarm robots operating as a crowd.
3. Literature review and survey papers not describing evaluation approaches.
4. Papers where the experimental setup was not clearly presented.

**Table 6:** Systematic review for robot task allocation. *Plus publications reviewed in Cano et al.* (2016b)

Reference	Category (cf. Korsah et al. (2013))	Search Space ($N_{max}$)	Allocation	Method
Kim et al. (2012)	ND–MR–ST–IA	$1.6 \times 10{^5}$	Dynamic/simulated	Auction, distributed
Sikora et al. (2017)	ND–MR–ST–TA	$1.7 \times 10{^5}$	Static–dynamic/actual	Sub-optimal, time limited, centralised
Prescott et al. (2006)	ID–SR–MT–IA	$1.6 \times 10{^2}$	Dynamic/actual	Neural networks, distributed
Szomiki (2015)	ID–SR–MT–IA	2.6 $\times \,10{^2}$	Dynamic/simulated	Agent, distributed
Girard et al. (2008)	ID–SR–MT–IA	4.4 $\times \,10{^4}$	Static/simulated	Neural networks, distributed
Franceschelli et al. (2013)	ID–SR–MT–IA	5.0 $\times \,10{^6}$	Static+dynamic/simulated	Gossip algorithm, centralised + distributed
Nam and Shell (2015)	XD–SR–ST–IA	2.7 $\times \,10{^1}$	Static/simulated	Ranking algorithm/hungarian method, centralised
Liu and Shell (2012)	XD–SR–ST–IA	2.2 $\times \,10{^2}$	Dynamic/actual	Hungarian algorithm, centralised
Guo and Zhang (2010)	XD–SR–ST–IA	2.1 $\times \,10{^4}$	Dynamic/simulated	AI/auction, distributed
Hu and Xu (2013)	XD–SR–ST–IA	4.7 $\times \,10{^4}$	Dynamic/simulated	Cooperative control, distributed
Nam and Shell (2015)	XD–SR–ST–IA	1.0 $\times \,10{^6}$	Static/actual	Ranking algorithm/hungarian method, centralised
Liu and Shell (2011)	XD–SR–ST–IA	3.4 $\times \,10{^6}$	Dynamic/simulated	Multi-level partitioning, distributed
Liu and Shell (2012)	XD–SR–ST–IA	2.7 $\times \,10{^7}$	Dynamic/simulated	Hungarian algorithm, centralised
Liu and Shell (2013)	*XD–SR–ST–IA*	*1.0* $\times \,{ 10}^{{ 9}}$	*Dynamic/simulated*	*Auction, distributed*
Liu and Shell (2012)	*XD–SR–ST–IA*	*1.0* $\times \,{ 10}^{ 12}$	*Dynamic/simulated*	*Swap, distributed*
Suemitsu et al. (2016)	XD–SR–ST–TA	1.0 $\times \,10{^2}$	Static/actual	Genetic algorithm, centralised
Chu and ElMaraghy (1993)	XD–SR–ST–TA	1.6 $\times \,10{^4}$	Static/actual	Heuristic, centralised
Caraballo et al. (2017)	XD–SR–ST–TA	8.0 $\times \,10{^4}$	Dynamic/simulated	Negotiation, distributed
Dias and Stentz (2002)	XD–MR–ST–IA	8.0 $\times \,10{^2}$	Static/simulated	Market-trading, distributed
Giordani et al. (2013)	XD–MR–ST–IA	7.5 $\times \,10{^5}$	Dynamic/simulated	Auction/hungarian method, distributed
Chen and Sun (2011)	XD–MR–ST–IA	6.3 $\times \,10{^3}$	Static+dynamic/actual	Coalition-based, distributed
Zhang and Parker (2013)	*XD–MR–ST–IA*	*7.4* $\times \,{ 10}^{{ 9}}$	*Dynamic/simulated*	*Heuristic, distributed*
Parker and Gini (2014)	XD–MR–ST–TA	1.0 $\times \,10{^4}$	Dynamic/simulated	Heuristic, distributed
Agarwal et al. (2015)	XD–MR–ST–TA	6.3 $\times \,10{^4}$	Dynamic/simulated	Coalition based, centralised
Miyata et al. (2002)	*XD–SR–MT–IA*	*3.2* $\times \,{ 10}^{{ 2}}$	*Static/actual*	*Priority-based, centralised*
Lagoudakis et al. (2004)	XD–SR–MT–IA	1.2 $\times \,10{^3}$	Dynamic/simulated	Auction, distributed
Lozenguez et al. (2013)	XD–SR–MT–IA	1.6 $\times \,10{^4}$	Dynamic/simulated	Auctions/markov decision, distributed
Sujit et al. (2006)	XD–SR–MT–IA	1.8 $\times \,10{^4}$	Dynamic/simulated	Negotiation, distributed
Zhao et al. (2016)	XD–SR–MT–IA	6.6 $\times \,10{^4}$	Dynamic/simulated	Heuristic, distributed
Luo et al. (2015)	*XD–SR–MT–IA*	*1.4* $\times \,{ 10}^{{ 6}}$	*Dynamic/simulated*	*Auction, distributed*
Nunes et al. (2012)	XD–SR–MT–IA	2.5 $\times \,10{^6}$	Static/simulated	Auction, distributed
Mosteo and Montano (2007)	XD–SR–MT–IA	5.2 $\times \,10{^6}$	Dynamic/simulated	Auction, distributed
Tolmidis and Petrou (2013)	XD–SR–MT–IA	7.2 $\times \,10{^7}$	Dynamic/simulated	Auction/genetic algorithm, centralised + distributed
Okamoto et al. (2011)	XD–SR–MT–IA	8.0 $\times \,10{^8}$	Dynamic/simulated	Peer-to-peer token passing, distributed
Balakirsky et al. (2007)	*XD–SR–MT–TA*	*2.0* $\times \,{ 10}^{{ 3}}$	*Dynamic/simulated*	*Auction, distributed*
Zorbas et al. (2016)	XD–SR–MT-TA	1.3 $\times \,10{^5}$	Static/simulated	Greedy heuristic, centralised

Italics represent to the original conference paper

Each paper was assessed for inclusion by two of the authors. Disagreements were arbitrated by a third author, at which point the decision was final. Additionally, to restrict the search scope we established quality criteria. As the extracted information relates to experimental setup and not results of any of the reviewed articles, no publication bias is expected and thus no measures were designed in this protocol to avoid it. On the other hand, only conferences included in the highest rank of three ranking systems were included (AMiner 2017; Wirtschaftsinformatik 2017; CORE 2017). By looking at three ranking systems we attempt to remove bias and error from the ranking method. The ranking systems were accessed online on 19 January 2017. For journal publications a 2 year JCR impact factor higher than 2 was selected as a qualitative criterion for selection.

The information extracted from each paper is:

1. Number of robots used in the experiment.
2. Total number of tasks to be allocated.
3. Number of tasks per robot.
4. Allocation method.

Allocation methods were categorised as static when they were performed ahead of time or dynamic when tasks were allocated in the duration of the experiment. Moreover, the methods were categorised as centralised or distributed. Each publication was then characterised based on the extended taxonomy found in Korsah et al. (2013). From this information the search space $N_{max}$ was calculated for each publication using Eq. 8, based on the largest experiment or maximum possible search space. This formula was simplified for auction and negotiation methods to:

10 $$\begin{aligned} N_{max} = { n \times \tau ^2 } \end{aligned}$$

where $\tau$ is the number of tasks that can be allocated, and *n* is the number of robots negotiating or bidding for tasks. Similarly, there is another simplified formula for Neural Network methods:

11 $$\begin{aligned} N_{max} = \prod _{ i \in layers } { nodes _i} \end{aligned}$$

where $$ layers $$ denote the layers of the Neural Network and $$ nodes _i$$ the number of nodes in the *i*th layer. Finally, when an explicit formula was presented in a publication that formula took precedence as long as it respected the constraints in Eq. 8.

As a result of this systematic survey, Table 6 presents the papers reviewed and a summary of information extracted. The selection of articles was performed 16–31 January 2017 with the information extraction and review of articles performed by 15 February 2017. Entries are sorted primarily by taxonomic classification, then by search space size. The work presented in the earlier sections of this paper is classified as XD–SR–MT–IA. Thus, by comparison, the search space of our work is several orders of magnitude larger than the maximum search space presented in related work in this category (Lagoudakis et al. 2004; Lozenguez et al. 2013; Luo et al. 2015; Miyata et al. 2002; Sujit et al. 2006; Zhao et al. 2016; Nunes et al. 2012; Mosteo and Montano 2007; Tolmidis and Petrou 2013; Okamoto et al. 2011).

### Comparative analysis

The purpose of the systematic survey was primarily to quantify the search space of prior robotic case studies. In this section, we provide a deeper comparison of our work with the most closely related previous research.

The first difference arises from the number of tasks and agents considered. Prior work based on the linear assignment problem (Pentico 2007) assumes a single task per agent (Nam and Shell 2015; Luo et al. 2015; Liu and Shell 2013, 2012). In our case, the number of tasks is equal to or greater than the number of agents (and the number of variants is greater still). A second point is related to the number of agents simultaneously completing tasks. In Chen and Sun (2011), Zhang and Parker (2013), Balakirsky et al. (2007) several agents are required, while in our work only one agent is completing each task. Another consideration is that our system is fully heterogeneous, i.e. all tasks and processors may be different. Some past work does assume heterogeneous tasks and multiple instances of every task (Miyata et al. 2002), but does not consider different variants of the same task, which is the principal addition to the problem here.

On the other hand, Aleti et al. (2013) provide a high-level general survey of software architecture optimisation techniques. In their taxonomy, our work is in the problem domain of design-time optimisation of embedded systems. We explore optimisation strategies that are both approximate and exact. We evaluate our work via both benchmark problems and a case study. In terms of the taxonomy in Aleti et al. (2013) our work is particularly wideranging.

Finally, Huang et al. (2012) consider the selection and placement of task variants for reconfigurable computing applications. They represent applications as directed acyclic graphs of tasks, where each task node can be synthesised using one of four task variants. The variants trade off hardware logic resource utilisation with execution time. Huang et al. use an approximate optimisation strategy based on genetic algorithms to synthesise the task graph on a single FPGA device.

To summarise, no existing work in the robotics field addresses all of the considerations that our proposal does, i.e. a constrained, distributed, heterogeneous system with more tasks than nodes and different variants for the tasks.

## Conclusion

We have addressed a unique generalisation of the task allocation problem in distributed systems, with a specific application to robotics. We advocate the use of task variants, which provide trade-offs between QoS and resource usage by employing different parameter configurations, and/or algorithms, and/or taking advantage of heterogeneous hardware. We have presented a mathematical formulation of variant selection and allocation, and evaluated three solution methods on system instances obtained from a robotics case study. We conclude that constraint programming is the best solution method, being very effective in selecting and allocating variants such that QoS is maximised and resource usage minimised. In addition, we find that our solutions methods translate well to real systems, providing a useful tool for the system architect. We also analysed two solution methods in anytime mode, concluding that constraint programming might be used in dynamic scenarios. Finally, we performed a comprehensive literature survey on prior case studies and found that the maximum search space of our case study is much larger than those in previous work. We believe future work on constraint programming can further extend the boundaries of what has been handled in this work.

## Footnotes

1 All the source code can be found online (White and Cano 2017).

2 Note that we simulate the QoS and CPU loads given the characterised values in Table 1. No physics/robot simulation was used.

3 Note that this paper is an extension of our previous work (Cano et al. 2016b).
